# An endogenous F-box protein regulates ARGONAUTE1 in *Arabidopsis thaliana*

**DOI:** 10.1186/1758-907X-1-15

**Published:** 2010-07-12

**Authors:** K Earley, MR Smith, R Weber, BD Gregory, RS Poethig

**Affiliations:** 1 Department of Biology, University of Pennsylvania, Philadelphia PA 19104, USA

## Abstract

ARGONAUTE1 (AGO1) mediates microRNA- and small interfering RNA-directed posttranscriptional gene silencing in *Arabidopsis thaliana*. Mutant alleles of *SQUINT *(*SQN*) slightly reduce AGO1 activity and have weak effects on shoot morphology. A screen for mutations that suppress the *sqn *phenotype produced loss-of-function mutations in the F-box gene *FBW2*. Mutations in *FBW2 *not only suppress *sqn *but also suppress many of the developmental phenotypes of weak, but not null, alleles of *AGO1 *by increasing AGO1 protein levels. Conversely, over-expression of FBW2 decreases the abundance of the AGO1 protein but not *AGO1 *messenger RNA, further indicating that FBW2 regulates AGO1 protein levels. *fbw2 *mutants have no obvious morphological phenotype, but display a reduced sensitivity to abscisic acid (ABA) that can be attributed to increased AGO1 activity. Our results indicate that FBW2 is a novel negative regulator of AGO1 and suggest that it plays a role in ABA signalling and/or response.

## Background

Argonaute proteins are core components of the RNA-induced silencing complex (RISC) [1-3]. These proteins use microRNAs (miRNAs) and/or small interfering RNAs (siRNAs) as guides to direct RISC to a specific site in target mRNAs, resulting in the cleavage or translational repression of these target mRNAs. Some Argonaute proteins also promote transcriptional repression through their effect on chromatin structure [2-4].

ARGONAUTE1 (AGO1) is one of 10 Argonaute proteins in *Arabidopsis thaliana *[2,5]. Genetic analyses [6-9], as well as the identification of the small RNAs that co-purify with AGO1 [10,11], indicate that AGO1 plays a central role in both miRNA and siRNA-mediated RNA silencing. Arabidopsis is exquisitely sensitive to the level of AGO1 activity, as evident from the broad range of phenotypes displayed by hypomorphic mutations of this gene [5,6,8,12]. In wild-type plants, the expression of AGO1 is maintained at a constant level by a negative feedback loop involving miR168. AGO1 is a target of miR168 and negatively regulates its own activity by promoting the activity and stability of miR168 [9,13] and by promoting the activity of siRNAs derived from the AGO1 transcript [14]. AGO1 activity is negatively regulated by PNH/ZLL/AGO10 [15] and positively regulated by SQUINT (SQN), the Arabidopsis orthologue of the protein chaperone, Cyclophilin-40 [12].

Null alleles of *SQN *have a morphological phenotype that is nearly identical to the phenotype of weak loss-of-function alleles of *AGO1 *[12]. In order to identify genes involved in *AGO1*-mediated processes, we screened for mutations that suppress the phenotype of *sqn-1*. This screen yielded several alleles of the F-box gene *FBW2*. Here we show FBW2 is a negative regulator of AGO1 and controls the sensitivity of plants to the hormone abscisic acid.

## Results

### Mutations in FBW2 rescue the sqn phenotype

Previously we found that SQN directly or indirectly promotes AGO1 activity [12]. In particular, we showed that the phenotype of loss-of-function alleles of *SQN *can be largely, if not entirely, explained by a reduction in the activity of AGO1.

**Figure 1:**
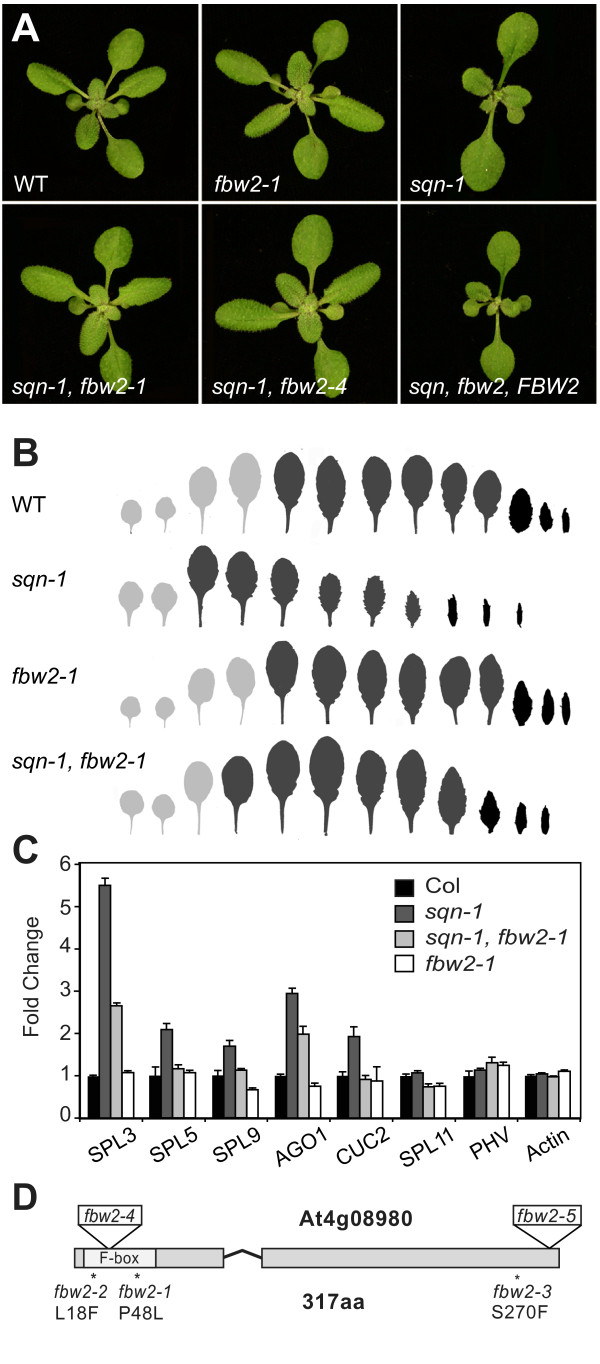
**Loss-of-function mutations of *FBW2 *suppress the phenotype of *sqn-1***. (A) An 18-day-old rosettes of wild-type (WT), *fbw2-1, sqn-1, sqn-1 fbw2-1, sqn-1 fbw2-4 *and *sqn-1 fbw2-1 *plants containing a genomic *pFBW2::FBW2-FLAG *construct. Rosettes are same magnification. (B) Leaf shape of successive juvenile (light grey) adult (dark grey) and cauline leaves (black) in WT, *fbw2-1, sqn-1 *and *sqn-1 fbw2-1 *plants. Juvenile leaves were defined by the absence of abaxial trichomes (*n *= 24; ± standard deviation). (C) Relative abundance of miRNA targets in various genotypes as measured by quantitative-real time polymerase chain reaction. Target genes were normalized to EIF4. Actin was used as a non-target control (± standard deviation). (D) The genomic structure of *FBW2 *and the amino acid changes produced by *fbw2-1, fbw2-2 *and *fbw2-3*. The locations of the T-DNA insertions in *fbw2-4 *(SALK_144548) and *fbw2-5 *(SALK_071588) are also illustrated.

In order to study the mechanism of this regulation, we screened for ethyl methanesulfate (EMS)-induced mutations that suppress the phenotype of the null allele, *sqn-1*. *sqn-1 *transiently delays leaf initiation, accelerates the juvenile-to-adult transition and produces aberrant spacing of flowers in the inflorescence and an increase in carpel number [16] (Figure 1A and 1B). Three allelic mutations that partially suppress all of these phenotypes were identified in this screen (Figure 1A, B and 1D). In addition to their effect on the morphological phenotype of *sqn-1*, these alleles decreased the expression of several miRNA-targeted genes (*SPL3, SPL5, SPL9, AGO1, CUC2*) previously shown to be over-expressed in *sqn-1 *[12] (Figure 1C). All three mutations had no obvious effect on shoot morphology or gene expression in the absence of *sqn-1 *(Figure 1A, B and 1C).

Using a map-based approach, we determined that this suppressor corresponds to *FBW2 *(*F-BOX WITH WD-40 2*) [17]. All three alleles change conserved residues in the predicted FBW2 protein (Figure 1D). Furthermore, a ~4,700-bp genomic construct expressing FBW2 under its native promoter (*pFBW2::FBW2-FLAG*) restored the original *sqn-1 *phenotype when transformed into *sqn-1 fbw2-1 *(Figure 1A). Two additional mutations of *FBW2 *were identified in the SALK collection of T-DNA insertions [18], SALK_144548 (*fbw2-4*) and SALK_071588C (*fbw2-5*; Figure 1D). Like the point mutations recovered in our screen, both of these mutations had no obvious morphological phenotype but nearly completely suppressed the phenotype of *sqn-1 *in double mutants (Figure 1A). A real time polymerase chain reaction (RT-PCR) of the *FBW2 *transcript in *sqn-1 fbw2 *double mutants revealed that *fbw2-2 *and *fbw2-3 *have no effect on the abundance of this transcript, *fbw2-1 *and *fbw2-5 *reduce, but do not eliminate, the transcript, and that *fbw2-4 *has no detectable FBW2 mRNA (Additional File 1: Figure S1A). We conclude that loss-of-function mutations of *FBW2 *suppress the phenotype of *sqn-1*.

*FBW2 *is predicted to encode a 317-amino acid protein with an N-terminal F-box domain [17] (Figure 1D). Although FBW2 was originally described as having a WD-40 domain [17], we found no evidence for the presence of a canonical WD-40 domain in this protein. A number of other predicted proteins in the Arabidopsis genome have sequence similarity to FBW2, but the similarity between these proteins is quite low (less than 32% identical), which suggests that FBW2 may be functionally unique (Additional File 1: Figure S1B). FBW2 is highly conserved in flowering plants (Additional File 1: Figure S1C), but is absent in the alga, *Chlamydomonas reinhardtii*. Interestingly, FBW2 has no strong sequence similarity to the Polerovirus P0 protein, an F-box protein that suppresses posttranscriptional gene silencing by destabilizing AGO1 [19,20].

### Loss of FBW2 rescues hypomorphic *ago *mutations

**Figure 2:**
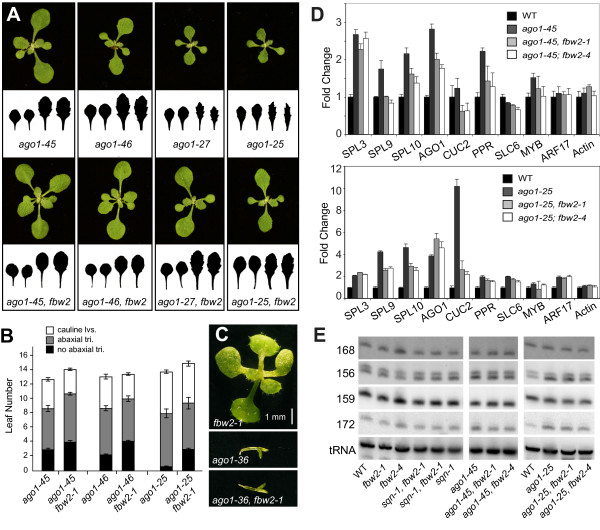
**Mutations in *FBW2 *suppress hypomorphic *ago1 *mutations**. (A) Eighteen-day-old rosettes of *ago1 *mutants and *ago1 fbw2-1 *double mutants. The shapes of leaves 1-4 are also illustrated. (B) The number of juvenile, adult and cauline leaves in *ago1 *and *ago1 fbw2-1 *double mutants (± standard deviation). (C) 14-day-old *fbw2-1*, *ago1-36 *and *ago1-36 fbw2-1 *double mutants grown on MS media. (D) Relative abundance of microRNA (miRNA) targets in various genotypes as measured by quantitative-real time polymerase chain reaction in 14-day-old (top) or 20-day-old (bottom) rosettes. Target genes were normalized to EIF4. Actin was used as a non-target control (± standard deviation). (E) Small RNA blots of low-molecular-weight RNA isolated from 14-day-old or 20-day-old rosettes probed with oligonucleotides complementary to specific miRNAs. Methionyl transfer RNA was used as a loading control.

Previous work from our laboratory suggested that the sole function of SQN is to promote the activity of AGO1 [12]. Given that *fbw2 *rescues null mutations of *SQN*, we recognized that FBW2 cannot function through SQN. A reasonable alternative hypothesis is that *fbw2 *mutations suppress *sqn-1 *by increasing the activity of AGO1. A prediction of this hypothesis is that *fbw2 *mutations should be capable of suppressing at least some hypomorphic alleles of *AGO1 *but should have no effect on the phenotype of a null allele. In order to test this hypothesis, we crossed *fbw2-1 *and *fbw2-4 *to 4 weak *ago1 *mutant alleles (*ago1-25, ago1-27, ago1-45*, and *ago1-46*) and the null allele, *ago1-36 *[8,12,21]. As predicted, *fbw2-1 *and *fbw2-4 *partially rescued the developmental and molecular phenotypes of all four hypomorphic *ago1 *mutations. Double mutants had a faster rate of leaf initiation, a later onset of abaxial trichomes, and larger and less serrated leaves than *ago1 *single mutant plants (Figure 2A and 2B). They also displayed a decrease in the expression of many of the miRNA-targeted genes upregulated in *ago1 *single mutants [9,12] (Figure 2D). In contrast, we observed no difference between the phenotype of *ago1-36 *single mutants and *ago1-36 fbw2-1 *double mutants (Figure 2C). These observations suggest that FBW2 either acts upstream of AGO1 or affects AGO1 activity.

The observed decrease in miRNA-regulated transcripts in *sqn fbw2 *and *ago1 fbw2 *compared with *sqn *and *ago1 *(Figure 1C and Figure 2D) could be explained by an increase in the accumulation of the miRNAs that target these transcripts for degradation. We did not favour this hypothesis because our genetic evidence suggests that FBW2 acts through AGO1, and *ago1 *hypomorphic alleles have limited effects on miRNA levels [9,12] (Figure 2E). Nevertheless, we compared the level of several miRNAs in *sqn fbw2 *and *ago1 fbw2 *double mutants with *sqn *and *ago1 *(Figure 2E). As predicted, *sqn-1 *and two hypomorphic *ago1 *alleles had weak or no effect miRNA levels and there was no significant difference between the miRNA levels in these single mutants and *sqn-1 fbw2 *and *ago1 fbw2 *double mutants (Figure 2E). Individually, *fbw2 *mutations also had no effect on miRNA levels (Figure 2E). Thus, the reduction in the abundance of miRNA-regulated transcripts in *sqn fbw2 *and *ago1 fbw2 *double mutants is not the result of an increase in miRNA expression.

### *fbw2 *mutations increase the abundance of AGO1

**Figure 3:**
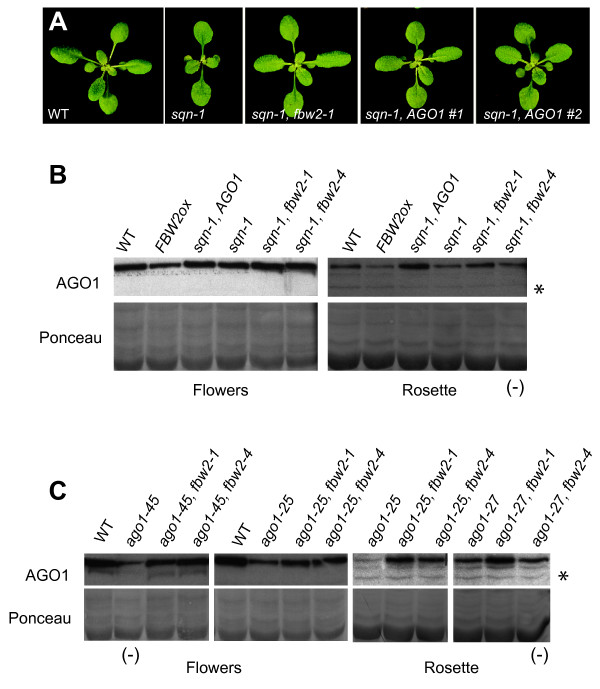
**Mutations in FBW2 suppress *sqn *and *ago1 *by increasing ARGONAUTE1 (AGO1)**. (A) Eighteen-day-old rosettes of WT, *sqn-1, sqn-1 fbw2-1*, and two independently isolated *sqn-1 *lines containing an *AGO1::FLAG-AGO1 *transgene. *sqn-1 AGO1::FLAG-AGO1 *transgenics resemble *sqn fbw2 *mutants. (B, C) Western blot of protein extracts from 14-day-old rosettes or floral buds probed with an anti-AGO1 antibody. Ponceau staining and a nonspecific band (*) were used as a loading control. (-) indicates samples that were slightly under-loaded.

**Figure 4:**
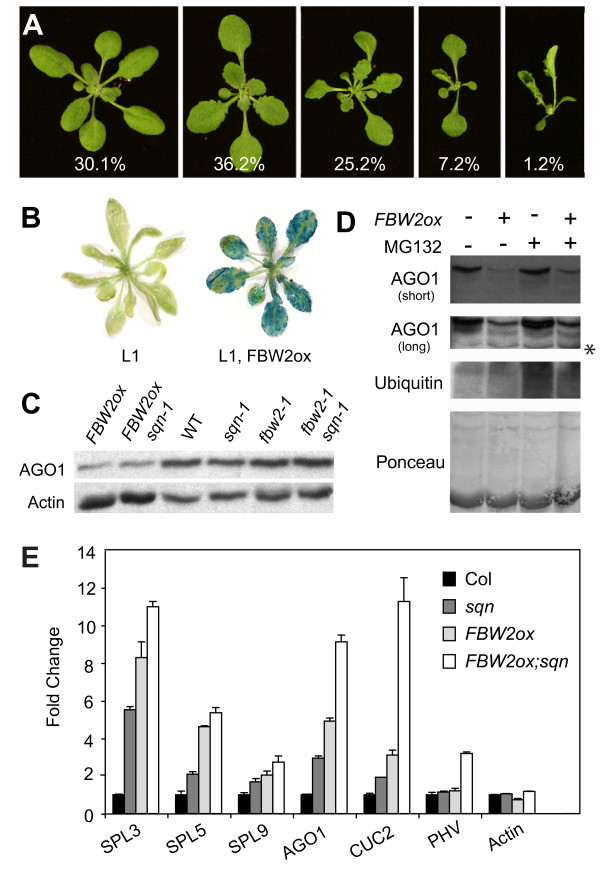
**Over expressing *FBW2 *decreases ARGONAUTE1 (AGO1) protein levels**. (A) Primary transformants containing a genomic *35S::FBW2 *(*FBW2ox*) transgene. The percentage of plants displaying each phenotype is indicated (n = 345 plants). (B) L1 plants with, and without, the *FBW2ox *construct. *FBW2ox *restores the expression of the *35S::GUS *transgene present in L1. (C) Western blot of protein extracts from 14-day-old seedlings probed with an anti-AGO1 antibody. AGO1 is reduced in plants containing *FBW2ox*. Actin was used as a loading control. (D) The proteasome inhibitor MG132 has no effect on AGO1 protein levels. Western blots of protein isolated from plants treated as indicated were probed with anti-AGO1 antibody. The lower anti-AGO1 blot is a longer exposure. The increased abundance of ubiquitinated proteins in MG132-treated plants indicates that the treatment was effective. Ponceau staining and a nonspecific band (*) were used as a loading control. (E) Abundance of miRNA targets in various genotypes as measured by quantitative-real time polymerase chain reaction. The abundance of these transcripts is significantly greater in *FBW2ox sqn-1 *than in the parental lines, suggesting that *sqn-1 *and *FBW2ox *operate independently to reduce AGO1 activity. Results were normalized to EIF4 (± standard deviation).

We reasoned that if *fbw2 *mutations suppress *sqn-1 *by increasing the activity of AGO1, it should be possible to replicate this effect by simply increasing the dose of AGO1. In order to test this hypothesis, we transformed a *pAGO1::FLAG-AGO1 *construct into *sqn-1*. This construct expresses a FLAG-AGO1 translational fusion under the regulation of the endogenous *AGO1 *promoter. Numerous *sqn-1 *plants expressing this construct had near-WT phenotypes (Figure 3A). Indeed, the phenotypes of several of these *sqn-1 pAGO1::FLAG-AGO1 *lines were essentially identical to *sqn-1 fbw2 *double mutants (Figure 3A). Western blots demonstrated that, as expected, *sqn-1 pAGO1::FLAG-AGO1 *plants had slightly more AGO1 protein than *sqn-1 *(Figure 3B). This result provides additional support for the conclusion that the phenotype of *sqn *mutations is a consequence of a reduction in AGO1 activity--a conclusion that was originally based entirely on the phenotypic similarity between *sqn *and *ago1 *mutations and the genetic interaction between these mutations [12]. It also supports the hypothesis that *fbw2 *suppresses *sqn-1 *by increasing the activity of AGO1. As a direct test of this hypothesis, we compared AGO1 protein levels in wild-type, *sqn-1, ago1-25, ago1-45*, and combinations of these mutations with *fbw2*. We found that *sqn-1 *produced a small but reproducible decrease in AGO1 in both 14-day old leaf tissue and floral tissue, and that *fbw2-1 *and *fbw2-4 *suppressed this effect; *sqn-1 fbw2 *double mutants had approximately the same amount of AGO1 as wild-type plants (Figure 3B and Figure 4C). Similar results were obtained with hypomorphic alleles of AGO1: *ago1-25 *and *ago1-45 *had reduced amounts of AGO1 protein compared to wild-type plants, and *fbw2-1 *and *fbw2-4 *corrected this defect (Figure 3C). We conclude that *fbw2 *suppresses the phenotype of *sqn-1*, *ago1-25 *and *ago1-45 *by increasing the abundance of AGO1.

**Figure 5:**
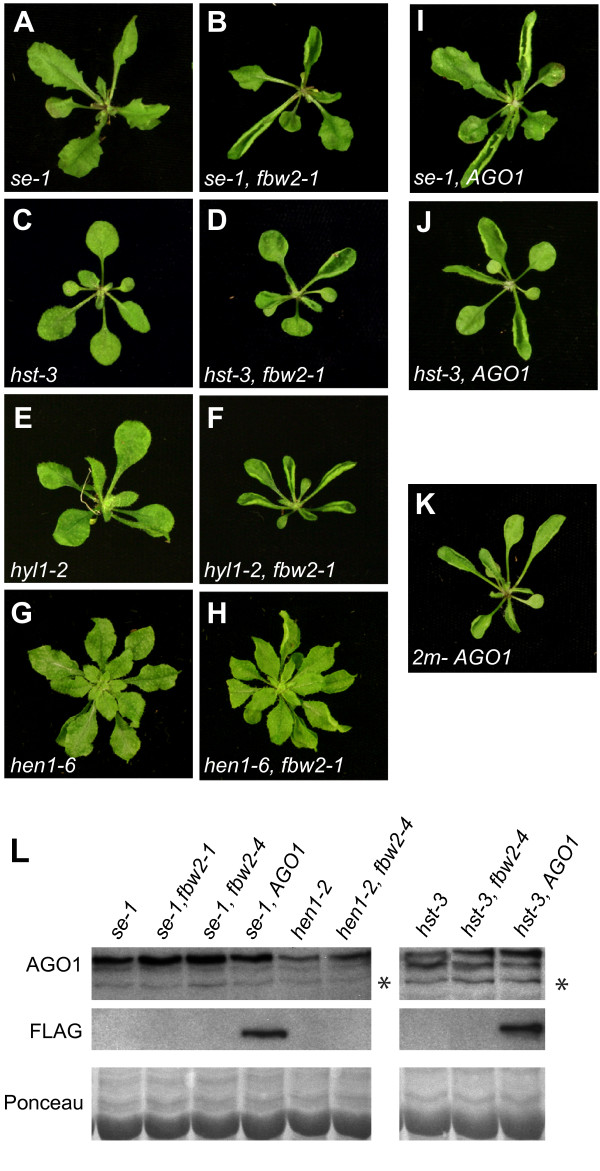
**Mutations in *FBW2 *enhance the *se-1, hst-3, hyl1-2 *and *hen1-6 *mutant phenotypes**. Twenty-one-day-old plants singly and doubly mutant for *fbw2-1 *and *hst-3 *(A and B) *se-1 *(C and D), or *hyl1-2 *(E and F), and 28-day-old *hen1-6 *and *hen1-6 fbw2-4 *plants (G and H). *hyl1-3 fbw2-1 *and *se-1 fbw2*-1 double mutants have phenotypes similar to *hyl1-3 *and *se-1 *plants transformed with *ARGONAUTE1::FLAG-AGO1 *(I and J) or WT plants containing the miR168 insensitive 2m-AGO1 construct (K). (L) Western blot of protein extracts from 14 day-old seedlings probed with an anti-AGO1 antibody. Ponceau staining and a nonspecific band (*) were used as a loading control.

We also examined the genetic interaction between *fbw2 *and several mutations that interfere with the biogenesis or stability of miRNAs, specifically *se-1 *(Figure 5A and 5B), *hst-3 *(Figure 5C and 5D), *hyl1-2 *(Figure 5E and 5F), and *hen1-6 *(Figure 5G and 5H). In every case, double mutants had stronger vegetative phenotypes than the single mutants. Although we were initially surprised by this result, we recognized that the phenotypes of these double mutants are remarkably similar to the phenotype of plants transformed with a miR168-resistant version of AGO1, which results in the over-expression of AGO1 (Figure 5K) [9,13]. In order to determine if this was a reasonable explanation for the effect of *fbw2*, we introduced *pAGO1::FLAG-AGO1 *into *se-1 *and *hst-3 *(Figure 5I and 5J) and also assayed AGO1 proteins levels in single and double mutant plants (Figure 5L). Many *hst-3 pAGO1::FLAG-AGO1 *and *se-1 pAGO1::FLAG-AGO1 *primary transformants had phenotypes that were almost identical to *hst-3 fbw2 *and *se-1 fbw2*, and strongly resembled plants containing miR168-resistant AGO1 mRNA constructs (Figure 5H - J). Consistent with this observation, western blots revealed increased levels of AGO1 in *se-1 fbw2*, *hen1-2 fbw2 *and *hst-3 fbw2 *double mutants compared to the single mutants (Figure 5L). These observations support the conclusion that *fbw2 *enhances the phenotypes of *se-1, hst-3, hyl1-2 *and *hen1-6 *by increasing AGO1 protein levels.

### Over-expression of FBW2 phenocopies *ago1 *mutants

F-box proteins are part of the E3 ubiquitin ligase complex, a protein complex that targets substrates for ubiquitin-mediated proteolysis through the 26S proteasome [22,23]. F-box proteins bind to unique substrates and thus provide specificity to the complex. Evidence that FBW2 (SKIP18) is a component of a E3 ubiquitin ligase complex is provided by the observation that it interacts with several different components of this complex in yeast two-hybrid assays [24]. We examined the effect of over-expressing FBW2 in transgenic plants, using the constitutively expressed Cauliflower Mosaic Virus 35S promoter (*35S::FBW2; *hereafter *FBW2ox*). This approach was suggested by the observation that over-expressing F-box proteins typically enhances the degradation of their protein targets [22,25-27]. The vast majority of the primary transformants we obtained in this experiment had developmental phenotypes strikingly similar to those of hypomorphic *ago1 *alleles, consistent with the hypothesis that FBW2 represses the activity of AGO1 (Figure 4A). In contrast, plants over-expressing three genes closely related to *FBW2*--*FBL9*, *FBL20*, and *SKIP1 *(Additional File 1: Figure S1B)--have no noticeable phenotype (data not shown), further suggesting that FBW2 is functionally unique.

In addition to promoting miRNA-mediated silencing, AGO1 is required for at least some forms of siRNA-mediated gene silencing, including the silencing of the *35S::GUS *transgene present in the L1 line [7]. In order to determine if FBW2 affects this aspect of AGO1 function, we transformed *FBW2ox *into L1 plants and assayed for GUS activity in families that were homozygous for the L1 transgene and segregating *FBW2ox*. In contrast to L1 plants--which had low levels of GUS activity--plants containing both *L1 *and *FBW2ox *had high levels of GUS activity (Figure 4B), like L1 *ago1 *mutants [7]. These results suggest that FBW2 affects both the miRNA- and siRNA-dependent activities of AGO1.

We tested to see if FBW2 promotes the degradation of AGO1 by examining the level of AGO1 protein in *fbw2-1 *mutants and *FBW2ox *transgenic plants. Plants over-expressing FBW2 had significantly less AGO1 than wild-type plants (Figures 2B and 4C-D). This decrease is not explained by an effect of FBW2 on the transcription of *AGO1 *because *AGO1 *mRNA--as well as the transcripts of several other miRNA-regulated genes--is actually elevated in *FBW2ox *relative to wild-type plants (Figure 4E). The increase in the transcript levels of these miRNA-regulated genes is consistent with the decreased level of AGO1 protein in *FBW2ox *plants; AGO1 promotes miRNA-mediated gene silencing and, thus, a decrease in the abundance of this protein should lead to an increase in the level of miRNA-regulated transcripts. In contrast to its effect on the AGO1 protein, *FBW2ox *had no effect on the abundance of the YFP-ZLL (AGO10) fusion protein (Additional File 2: Figure S2A)[28].

In order to determine if the FBW2-mediated decrease in AGO1 is proteasome dependent, we assayed AGO1 protein levels in the presence of the proteasome inhibitor MG132 [29]. Although treatment with MG132 produced a general decrease in protein degradation (Figure 4D), it had no effect on AGO1 protein levels in both wild-type and *FBW2ox *plants (Figures 4D and S2B). This result suggests that the *35S::FBW2*-mediated decrease in AGO1 is proteasome-independent and also indicates that AGO1 may be the target of an, as yet, unidentified proteasome-dependent degradation pathway. Interestingly, destabilization of AGO1 by the viral F-box protein P0 is also insensitive to MG132 [19].

Although *fbw2 *produces an observable increase in the amount of AGO1 protein in genetic backgrounds in which miRNA activity is compromised (Figures 3B and 3C, 4C and 5L), we were unable to detect a significant increase in AGO1 protein in *fbw2 *single mutants (Figure 4C). We suspect this is because the miR168-dependent feedback mechanism that regulates AGO1 expression [13] partially corrects for slight increases in the level of this protein in *fbw2 *mutants. Mutations that interfere with the activity of miR168 (for example, *sqn, ago1*and *hen1*) disrupt this feedback mechanism, thereby making AGO1 more susceptible to other factors that regulate its expression.

### *fbw2 *is hyposensitive to abscisic acid (ABA)

Although we were unable to observe an increase in AGO1 protein in *fbw2 *single mutants, these mutants have a phenotype that is indicative of an increase in AGO1. Mutations that interfere with miRNA biogenesis - including *hst*, *se*, *hyl1*, *dcl1 *and *hen1- *confer hypersensitivity to the phytohormone ABA [30,31]. These mutations inhibit both seed germination and root elongation in the presence of low levels of ABA, probably due to the misregulation of the miR159 targets *MYB101 *and *MYB33 *[32]. We reasoned that if *fbw2 *mutations increase AGO1 levels this should result in increased miRNA activity and produce the opposite phenotype, namely, hyposensitivity to ABA. Indeed, this is what we found.

**Figure 6:**
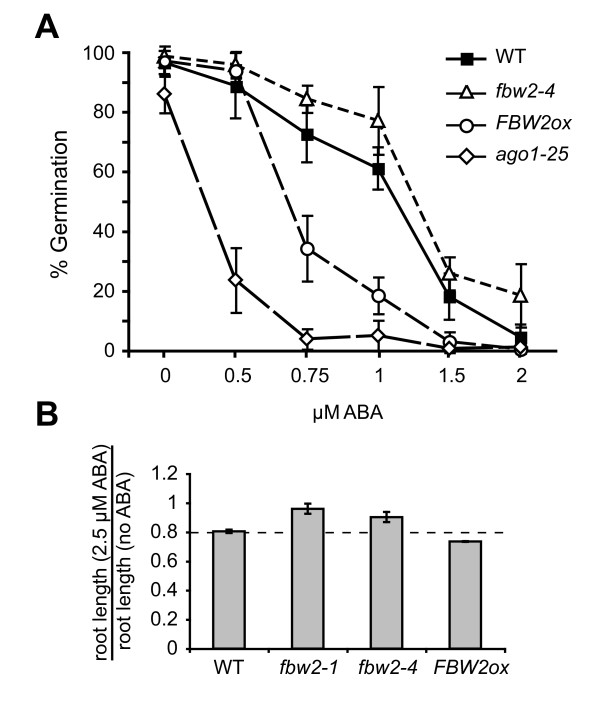
**FBW2 is required for a proper abscisic acid (ABA) response**. (A) The percent germination of WT, *fbw2-4, ago1-25 *and *FBW2ox *seeds in the presence of increasing concentrations of ABA (± standard deviation). (B) The effect of ABA on root elongation, reported as the ratio of root length in the presence of 2.5 μm ABA to root length in the absence of ABA (± standard deviation).

Seeds of various genotypes were grown on varying concentrations of ABA and scored for germination after 5 days. *ago1-25 *and *FBW2ox *were hypersensitive to ABA, with *ago1-25 *showing the greatest response. In the presence of ABA, both of these AGO1-deficient genotypes produced a significant decrease (*P *< 0.001 at 0.75 μM ABA) in seed germination relative to wild-type plants (Figure 6A); *FBW2ox *also displayed a slightly enhanced sensitivity to ABA in a root elongation assay (Figure 6B). Thus, AGO1 is required for a normal ABA response. In contrast, *fbw2 *mutants displayed a significantly increased rate of germination (*P *< 0.01 at 0.75 μM ABA) and increased rate of root elongation in the presence ABA (*P *< 0.03; Figure 6A and 6B). This result provides additional support for the conclusion that FBW2 normally represses the activity of AGO1 and reveals a physiological function for FBW2.

## Discussion

The results presented here demonstrate that FBW2 is a negative regulator of AGO1. We show that loss of FBW2 suppresses the phenotype of mutations that reduce the activity of AGO1 and that this effect is associated with an increase in AGO1 protein levels. Conversely, over-expressing FBW2 produces an *ago1 *loss-of-function phenotype and leads to a decrease in the level of AGO1. Although a loss of FBW2 does not produce a major change in the abundance of AGO1 in an otherwise wild-type background, the reduced sensitivity of *fbw2 *mutants to ABA is consistent with a slight increase in AGO1 activity. Previous studies have demonstrated that Arabidopsis is hypersensitive to changes in AGO1 activity [8,9,12] so it would not be surprising if even a minor change in the abundance of this protein is biologically significant. Over-expressing FBW2 did not produce an observable decrease in the Argonaute protein AGO10/ZWL/PNH, suggesting that FBW2 may act specifically on AGO1.

The simplest and most direct way in which FBW2 could regulate AGO1 is by binding directly to this protein, thereby directing it to a proteasome-independent degradation pathway. However, we have been unable to detect a direct interaction between FBW2 and AGO1 (data not shown). Consequently, we cannot eliminate the possibility that FBW2 acts indirectly, through its effect on a protein required for AGO1 stability. This protein cannot be SQN because *fbw2 *mutations rescue the phenotype of the null allele, *sqn-1*. Furthermore, over expressing FBW2 produces a much more severe phenotype than that of *sqn *null alelles. Recent work suggests that the viral F-box protein, P0, acts by targeting an unknown component of the AGO1 RISC complex, leading to the destabilization and degradation of AGO1 [33]. It may be that FBW2 regulates this same protein or another protein within the RISC complex. Loss-of-function mutations in this hypothetical FBW2-regulated factor would be expected to have the same phenotype as FBW2ox plants, such as an *ago1 *loss-of-function phenotype. Extensive screens for genes required for miRNA and siRNA biogenesis and activity by us and others have produced many loss-of-function alleles of AGO1 but, with the exception of *sqn*, have yet to reveal other *ago1*-like mutants. Determining the identity of this unknown protein (if it exists) may require a biochemical approach.

The activity of AGO1 in Arabidopsis is regulated by a variety of different mechanisms that act together to maintain the expression of this protein at a constant level [9-11,13,15]. This is critical because both an increase and a decrease in the abundance of AGO1 have significant effects on plant development. An important component of this homeostatic mechanism is the negative regulation of AGO1 by miR168 [9,13]. miR168 represses AGO1 in an AGO1-dependent fashion: a decrease in the activity of AGO1 leads to a decrease in miR168 activity and a subsequent increase in AGO1 expression, whereas an increase in AGO1 activity has the opposite effect. We believe this feedback loop is responsible for the observation that *fbw2 *mutations individually have no major effect on AGO1 because these same mutations elevate AGO1 protein levels in combination with mutations that interfere with miRNA biogenesis or activity.

Such finely tuned posttranscriptional regulation of an Argonaute protein is not unique to AGO1. The stability and sub-cellular localization of the mammalian protein Ago2 are influenced by hydroxylation and phosphorylation [34,35], while the turnover of Ago2 is controlled by an E3 ubiquitin ligase [36]--a mechanism that may be quite similar to the mechanism we propose here. Furthermore, Ago2 is post-translationally controlled by a variety of environmental and developmental cues, which operate via well-defined pathways [34-36]. It would not be surprising if environmental and developmental signals also play important roles in the regulation of AGO1. The observation that *fbw2 *has little, or no effect, on plant morphology, but decreases the sensitivity of plants to ABA, is relevant in this case. Among other things, ABA regulates the response to water stress. Therefore, the effect of *fbw2 *on ABA sensitivity suggests that changes in AGO1 activity may underlie the response to this and other environmental signals. The potential involvement of FBW2 in such regulatory pathways is an interesting subject for future studies.

## Conclusion

Our results demonstrate that FBW2 is a negative regulator of AGO1 and acts by destabilizing this protein. Although we are unable to determine whether FBW2 destabilizes AGO1 directly or via an effect on an as yet unknown protein, these results add yet another layer of control to the already complex mechanism responsible for AGO1 homeostasis. Loss of FBW2 affects the sensitivity of plants to the growth regulator ABA, suggesting a possible role for FBW2 in hormone response pathways.

## Methods

### Genetic stocks and growth conditions

Unless otherwise noted, all mutations described in this paper are in the Columbia background. *fbw2-1*, *fbw2-2 *and *fbw2-3 *were identified in M2 families of EMS-mutagenized *sqn-1 *plants. Primers for genotyping can be found in Additional File 3: Table S1. *ago1-45 *and *ago1-46 *have been previously described [12]. *ago1-36 *plants containing pGreen0229 *AGO1::FLAG-AGO1 *were provided by D Baulcombe (University of Cambridge, Cambridge, UK) [21]. *ago1-25*, *ago1-27*, *2m-AGO1 *lines and L1 lines were provided by H Vaucheret (Institut National de la Recherche Agronomique, Versailles, France) [7,8]. Plants containing YFP-AGO10 (*pZLL::YFP-ZLL*) in the Ler background were obtained from T Laux [28]. *fbw2-4 *(SALK_144548), *fbw2-5 *(SALK_071588), *hen1-2 *(SALK_090960), *hyl1-2 *(SALK_064863) and *se-1 *were obtained from the Arabidopsis Biological Resource Center http://abrc.osu.edu/[18]. Unless otherwise noted, plants were grown on Farfard soil at 23°C under 16 h fluorescent illumination. Wild-type or *fbw2 *plants segregating *ago1-36 *were grown on Murashigi and Skoog (MS) plates (0.5% MS, 1% sucrose, 0.8% agar pH 5.7). GUS assays were performed as previously described [37].

### RT-PCR

Total RNA was isolated using TRIzol (Invitrogen, CA, USA) from total above-ground tissue of 14-day-old seedlings. RNA was immobilized on RNeasy Plant Columns (Qiagen, CA, USA) and treated with DNAse (Qiagen). cDNA was amplified from 1-4 ug of RNA using Superscript II (Invitrogen). Quantitative RT PCR was performed using Sybr Green PCR Master Mix (Applied Biosystems, CA, USA) on a StepOnePlus™ RT-PCR System (Applied Biosystems). *FBW2 *RT Primers are described in Additional File 3: Table S1. Primers for RT-PCR of miRNA targets were previously described [12]. Transcript levels were normalized against *EIF4 *levels in all experiments.

### RNA blots

RNA blots were generated and analysed as previously described [12,38]. Briefly, total RNA was isolated using TRIzol (Invitrogen) from total above-ground tissue of 14-day-old seedlings. High molecular weight RNA was removed by precipitating with PEG-8000 (5%) and NaCl (500 mM). Low molecular weight (LMW) RNA was subsequently precipitated with 300 mM NaOAc and 2 vol 100% EtOH and washed with 70% EtOH. LMW RNA was separated on an 8 M urea/15% denaturing polyacrylamide gel and was transferred to a Hybond N membrane (Amersham Pharmacia, NJ, USA). Oligonucleotide probes were labelled using T4 polynucleotide kinase (New England Biolabs, MA, USA) with [γ -32P]-ATP at 40°C in ULTRAhyb-oligo hybridization buffer (Ambion, CA, USA). Membranes were hybridized with oligonucleotide probes complementary to specific miRNA targets.

### Western blot and immunoprecpitation experiments

Fourteen-day-old, 20-day-old seedlings, or floral buds were ground using liquid nitrogen and resuspended in 1:3 w/v extraction buffer [20 mM Tris pH 7.5, 300 mM NaCl, 5 mM MgCl_2_, 1× Protease Inhibitor Cocktail (Sigma, MO, USA) 1 mM PMSF, 1 mM DTT]. Equal amounts of soluble protein were separated on an 8.5% SDS-PAGE gel, transferred to a nitrocellulose membrane and membranes were blocked [tris buffered saline with tween (TBS-T) with 5% milk]. Anti-AGO1 (1:500; antibody provided by Y Qi and Xioafeng Cao, National Institute of Biological Sciences, Beijing, China), anti-FLAG monoclonal (1:2000) (Sigma - F1804), anti-FLAG HRP conjugated (1:2000; Sigma - A8592), anti-GFP (Invitrogen - A-6455), anti-cMyc rabbit (1:2000; Sigma - C3956), were incubated overnight in TBS-T + 5% milk at 4°. Anti-actin (1:10,000) (Sigma - A0480) was incubated at room temperature for 2 h.

### Constructs

The *FBW2 *coding or *FBW2 *genomic region was amplified by PCR using the *FBW2 *primers (Additional File 3: Table S1). PCR products were TOPO cloned into pENTR-D TOPO (Invitrogen). The *FBW2 *coding and the 4701-base pair genomic *FBW2 *genomic region were recombined into pEG100 (35S) and pEG302 (FLAG), respectively [39]. For *AGO1::FLAG-AGO1 *constructs, genomic *AGO1::FLAG-AGO *was amplified from the pGreen0229 FLAG-AGO1 construct using AGO1 F and R primers (Additional File 3: Table S1) [21]. PCR products were cloned into pENTR-D TOPO vector and recombined into the Cambia 3301 vector containing a GATEWAY cassette. All constructs were transformed using the Floral Dip technique [40].

### ABA experiments

Sterilized seeds were plated on 1% sucrose LS plates containing 0, 0.5, 0.75, 1.0, 1.5 or 2.0 μM ABA, imbibed for 3 days in the dark at 4°C and grown for 5 days under long day conditions (16 h light, 8 h dark). Plants were scored for greening of the cotyledons. Experiments were repeated at least three times. In order to determine the effect of ABA on root growth, sterilized seeds were plated vertically on 1% sucrose LS plates, imbibed for 3 days, and grown for 5 days under long day conditions (16 h light, 8 h dark). Seedlings of equal size were then transplanted to vertical plates containing either 0 μM or 2.5 μM ABA, and grown for 5 days or more under long day conditions (16 h light, 8 h dark). Primary root length was measured for each plant and root inhibition was determined as the average root length of plants grown at 2.5 μM ABA relative to the average root length of plants gown at 0 μM ABA.

## Abbreviations

ABA: abscisic acid; AGO1: ARGONAUTE1; EMS: ethyl methanesulfonate; mRNA: messenger RNA; miRNA: microRNA; RISC: RNA-induced silencing complex; RT-PCR: real time polymerase chain reaction; siRNA: small interfering RNA; SQN: SQUINT; TBS-T: tris buffered saline tween.

## Competing interests

The authors declare that they have no competing interests.

## Authors' contributions

KWE performed most of the work described in this paper and co-wrote the manuscript with RSP. MRS performed the *sqn *suppressor screen. MRS and KWE mapped *FBW2*; RW performed the ABA experiments under the guidance of BDG.

## Supplementary Material

Additional file 1 **Figure S1**. (A) Gene structure of FBW2 showing the location of primers used in real time polymerase chain reaction and the abundance of FBW2 messenger RNA in *sqn-1 *and mutants doubly mutant for *sqn-1 *and different alleles of *fbw2*. All target genes were normalized to EIF4. (B) Alignments of the *Arabidopsis *F-box genes most closely related to At4g08980 (FBW2): At4g05497 (FBW9), At4g05460 (FBL20), At5g57900 (SKIP1). (C) Alignments of the amino acid sequences of FBW2 and its predicted orthologs in other flowering plants. All alignments were made using the following assembled contigs from PlantGDB unless otherwise noted: *Citrus sinensis *(PUT-157a-Citrus_sinensis-6728477), *Gossypium raimondii *(PUT-157a-Gossypium_raimondii-11427), *Glycine max *(PUT-161a-Glycine_max-76896), *Oryza sativa Japonica *(Genbank AK070008), *Asparagus officinalis *(Genbank CV288647), *Vitis vinifera *(PUT-157a-Vitis_vinifera-16230), *Nicotiana tabacum *(PUT-163a-Nicotiana_tabacum-55992858), *Lactuca sativa *(PUT-157a-Lactuca_sativa-30499), *Pinus taeda *(PUT-157a-Pinus_taeda-79282550). Click here for file

Additional file 2 **Figure S2**. (A) Western blot comparing levels of ARGONAUTE1 (AGO1) and yellow fluorescent protein-AGO10 in plants with and without *FBW2ox *constructs. Anti-AGO1 and anti-GFP antibodies were used to probe each blot. Ponceau staining was used as a loading control. (B) Levels of AGO1-FLAG do not decrease in plants treated with the protease inhibitor MG132. Blots were probed with anti-FLAG monoclonal antibody or anti-ubiquitin antibody. The anti-ubiquitin antibody demonstrates an overall increase in ubiquitination in MG132 treated plants, as expected from a decrease in proteasome activity. Click here for file

Additional file 3 **Table S1**. Polymerase chain reaction primers used in this study. Click here for file
