# Supplementary material for: An endogenous F-box protein regulates ARGONAUTE1 in Arabidopsis thaliana
Source: Silence. 2010 Jul 12;1:15. doi: 10.1186/1758-907X-1-15 (PMC2914764; doi:10.1186/1758-907X-1-15)
Supplement: Additional file 1 — Figure S1. (A) Gene structure of FBW2 showing the location of primers used in real time polymerase chain reaction and the abundance of FBW2 messenger RNA in sqn-1 and mutants doubly mutant for sqn-1 and different alleles of fbw2. All target genes were normalized to EIF4. (B) Alignments of the Arabidopsis F-box genes most closely related to At4g08980 (FBW2): At4g05497 (FBW9), At4g05460 (FBL20), At5g57900 (SKIP1). (C) Alignments of the amino acid sequences of FBW2 and its predicted orthologs in other flowering plants. All alignments were made using the following assembled contigs from PlantGDB unless otherwise noted: Citrus sinensis (PUT-157a-Citrus_sinensis-6728477), Gossypium raimondii (PUT-157a-Gossypium_raimondii-11427), Glycine max (PUT-161a-Glycine_max-76896), Oryza sativa Japonica (Genbank AK070008), Asparagus officinalis (Genbank CV288647), Vitis vinifera (PUT-157a-Vitis_vinifera-16230), Nicotiana tabacum (PUT-163a-Nicotiana_tabacum-55992858), Lactuca sativa (PUT-157a-Lactuca_sativa-30499), Pinus taeda (PUT-157a-Pinus_taeda-79282550). [file 1758-907X-1-15-S1.PDF]

Sup. Figure 1

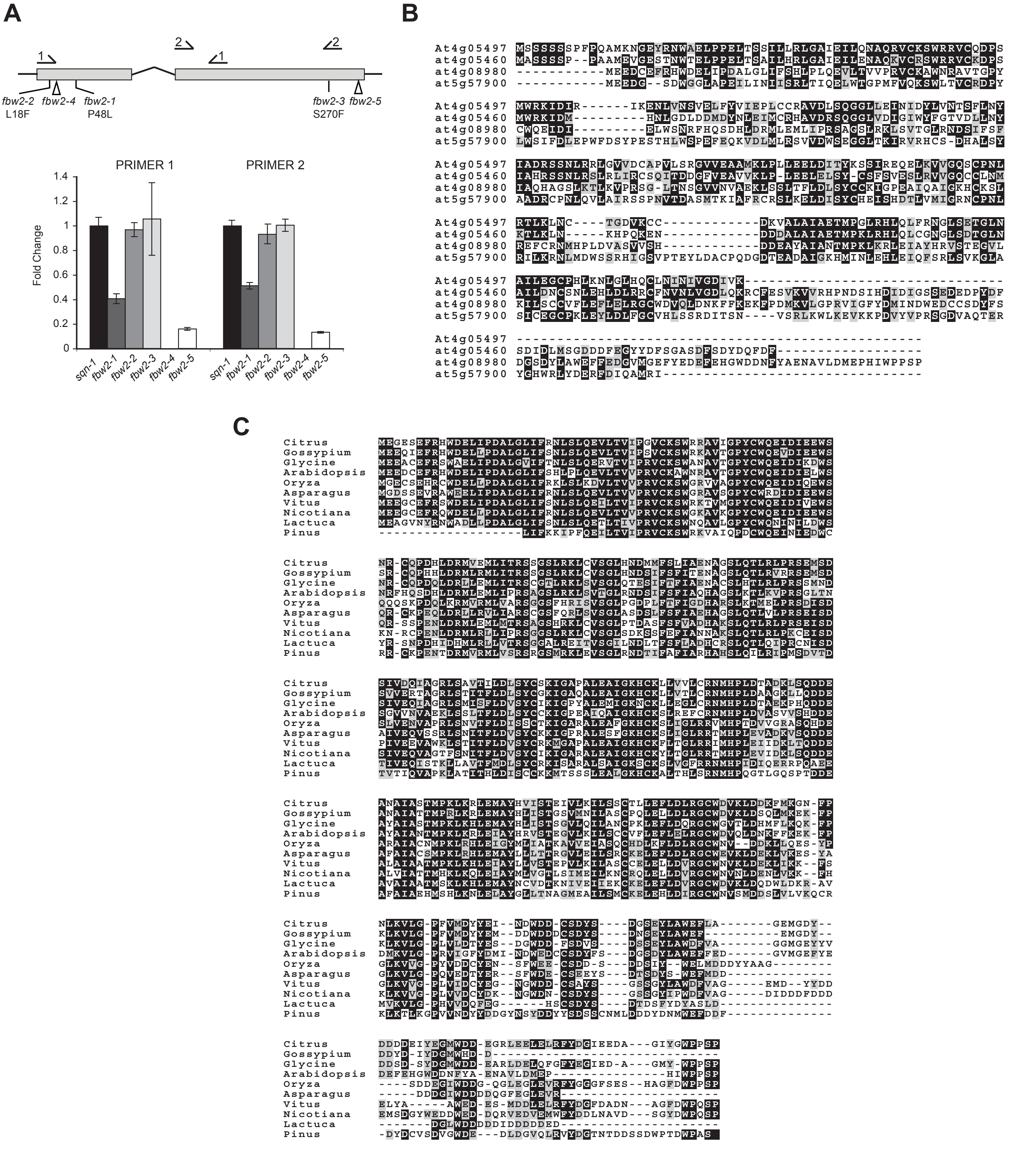
A
1
2
1
2
fbw2-2
L18F
fbw2-4
fbw2-1
P48L
fbw2-3
S270F
fbw2-5
PRIMER 1
PRIMER 2
Fold Change
1.4
1.2
1
0.8
0.6
0.4
0.2
0
sqn-1
fbw2-1
fbw2-2
fbw2-3
fbw2-4
fbw2-5
B
At4g05497
at4g05460
at4g08980
at5g57900
C
Citrus
Gossypium
Glycine
Arabidopsis
Oryza
Asparagus
Vitus
Nicotiana
Lactuca
Pinus
