# Supplementary figures and images for: An endogenous F-box protein regulates ARGONAUTE1 in Arabidopsis thaliana
Source: Silence. 2010 Jul 12;1:15. doi: 10.1186/1758-907X-1-15 (PMC2914764; doi:10.1186/1758-907X-1-15)

Sup. Figure 2

**A**

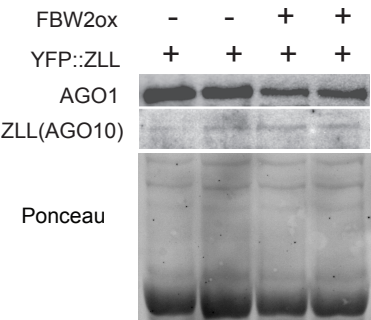

**B**

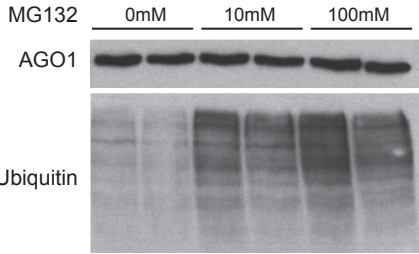

Supplement: Additional file 2 — Figure S2. (A) Western blot comparing levels of ARGONAUTE1 (AGO1) and yellow fluorescent protein-AGO10 in plants with and without FBW2ox constructs. Anti-AGO1 and anti-GFP antibodies were used to probe each blot. Ponceau staining was used as a loading control. (B) Levels of AGO1-FLAG do not decrease in plants treated with the protease inhibitor MG132. Blots were probed with anti-FLAG monoclonal antibody or anti-ubiquitin antibody. The anti-ubiquitin antibody demonstrates an overall increase in ubiquitination in MG132 treated plants, as expected from a decrease in proteasome activity. [file 1758-907X-1-15-S2.PDF]
