# Supplementary material for: An endogenous F-box protein regulates ARGONAUTE1 in Arabidopsis thaliana
Source: Silence. 2010 Jul 12;1:15. doi: 10.1186/1758-907X-1-15 (PMC2914764; doi:10.1186/1758-907X-1-15)
Supplement: Additional file 3 — Table S1. Polymerase chain reaction primers used in this study. [file 1758-907X-1-15-S3.DOC]

Table S1: Primers used in this study.

| **Primers for Constructs** | | | |
| --- | --- | --- | --- |
| Primer name | Primer Sequence (5' to 3') | Enzyme (if required) | Percentage Gel |
| cDNA FBW2 F | CACCATGGAAGAAGATTGCGAGTTTCG |  | 1% |
| cDNA FBW2 R | TCATGGAGATGGTGGCCAAATATGC |  |  |
| genomic FBW2 F | CACCCAAATTTCCACACTCCCTTCTCTGAACAG |  | 1% |
| genomic FBW2 R | TGGAGATGGTGGCCAAATATGC |  |  |
| FLAG-AGO1 F | CACCCGGGTGACAGCCACCACATTCCTAAAG |  | 1% |
| FLAG-AGO1 R | GGCGATCGCAAACGCATGAAATCAGTGAGAG |  |  |
| **Primers for RT PCR** | | | |
| FBW2 1 F | CACCATGGAAGAAGATTGCGAGTTTCG |  |  |
| FBW2 1 R | CCCGAATTAGTCAGACCACTTCTTGG |  |  |
| FBW2 2 F | GCGAAAACTCTCCGTTACTGGCCTTCG |  |  |
| FBW2 2 R | GTTATCAAGTTGCACGTCCCAACAGCC |  |  |
| **Primers for Genotyping** | | | |
| fbw2-1 (1007) | CACCATGGAAGAAGATTGCGAGTTTCG | Ava II | 2% |
|  | CCCGAATTAGTCAGACCACTTCTTGG |  |  |
| fbw2-2 (1336) | GACATTGGGATGAGTTGATTCCAGAAGCT | Hind III | 4% |
|  | GGTTACTCCACAGCTCAATGTCAATC |  |  |
| fbw2-3 (1258) | TGGAGATGGTGGCCAAATATGC | Hpy 188 I | 4% |
|  | GGATTGCTGCACAGATTACTTCACAGATGGG |  |  |
| fbw2-4 (salk_144548) | CTTGTTCTTGCTCATAGATTTTAGCTGTGG |  | 1% |
|  | CCCCTAATACATTCTATGCCTATAACC |  |  |
|  | CCCCTAATACATTCTATGCCTATAACC |  | 1% |
|  | ATTTTGCCGATTTCGGAAC |  |  |
| fbw2-5 (salk_071588c) | CGGTTAGAGATAGCTTACCACCG |  | 1% |
|  | GTTTCTTATACATCAACTTGACATCTCCAATTC |  |  |
|  | GTTTCTTATACATCAACTTGACATCTCCAATTC |  | 1% |
|  | ATTTTGCCGATTTCGGAAC |  |  |
| ago1-46 | TGATGTCTCTGGCTCCATGTAGAAGCTAG | Nhe I | 4% |
|  | TGCAAGATGCACACGCTCAGTTTC |  |  |
| ago1-45 | TGAGCCATGGTCTCGGATGTTTCA | SphI | 4% |
|  | GAGACTATGCCGAGTTCAGTCTCACGCATG |  |  |
| ago1-27 | TGCAAGATGCACACGCTCAGTTTC | Bann II | 4% |
|  | ACTCAGCAGTAGAACATGACACGC |  |  |
| sqn-1 | TCTGAGAGTAAATCAAGGTCAAA | Bsl I | 4% |
|  | GAAAGCCCAGCTGCCTTATCTTG |  |  |
